# Evaluating Educational Patterns and Methods in Infant Sleep Care: Trends, Effectiveness, and Impact in Home Settings—A Systematic Review

**DOI:** 10.3390/children11111337

**Published:** 2024-10-31

**Authors:** Maria Aggelou, Dimitra Metallinou, Maria Dagla, Victoria Vivilaki, Antigoni Sarantaki

**Affiliations:** Department of Midwifery, Faculty of Health and Care Sciences, University of West Attica, Egaleo, 12243 Athens, Greecemariadagla@uniwa.gr (M.D.); vvivilaki@uniwa.gr (V.V.); esarantaki@uniwa.gr (A.S.)

**Keywords:** infant sleep, safe sleep practices, parental education, home-based interventions, SIDS prevention

## Abstract

Background: Sleep care is crucial for the health and development of infants, with proper sleep patterns reducing the risk of sudden infant death syndrome (SIDS) and other sleep-related incidents. Educational interventions targeting caregivers are essential in promoting safe sleep practices. Methods: This systematic review adhered to PRISMA guidelines, searching databases such as PubMed, MEDLINE, Scopus, and the Cochrane Library. Inclusion criteria focused on studies involving home-based interventions for infants aged 0–12 months, including parental education and behavioral interventions. Exclusion criteria included studies in clinical settings and non-peer-reviewed articles. Data extraction and synthesis were performed by two independent reviewers, using a narrative approach to categorize interventions and outcomes. Results: Twenty-three studies met the inclusion criteria. Key findings indicate that home-based educational interventions, including hospital-based programs, home visits, and mobile health technologies, significantly improve parental knowledge and adherence to safe sleep practices. These interventions also enhance parental satisfaction and contribute positively to infant health outcomes. Conclusions: Educational interventions have demonstrated effectiveness in promoting safe sleep practices among caregivers, particularly in home settings. These interventions, including hospital-based programs, home visits, and digital tools, improve parental knowledge, adherence to guidelines, and overall satisfaction. The impact is evident in the reduction of unsafe sleep behaviors and enhanced infant health outcomes. However, variability in the intervention methods and delivery, cultural contexts, and geographic focus suggest a need for more tailored, long-term, and comprehensive studies. Future research should standardize outcome measures and assess the sustained impact of these educational strategies on infant sleep patterns and caregiver practices over time. This will provide deeper insights into the trends and long-term effectiveness of educational patterns and methods in diverse home environments.

## 1. Introduction

Sleep care is a critical aspect of infant health, directly influencing growth, development, and overall well-being. Establishing appropriate sleep patterns in neonates is pivotal in their physical and cognitive development, serving as a cornerstone for healthy developmental trajectories. Conversely, sleep disturbances in this age group can lead to significant challenges, impacting both the infant and the family. Poor sleep can affect an infant’s immune function, mood regulation, and cognitive processes while also contributing to parental stress and fatigue, potentially impairing the caregiver’s ability to provide optimal care [1].

Educational interventions targeting parents and caregivers are instrumental in promoting optimal sleep patterns in neonates. These interventions frequently encompass hospital-based education, home visiting programs, and parental workshops designed to promote safe sleep practices, establish consistent sleep routines, and mitigate the risk of sleep-related incidents such as sudden infant death syndrome (SIDS) [2]. The effectiveness of these interventions varies, influenced by factors such as the delivery method, cultural considerations, and the specific needs of the family [3].

Hospital-based educational programs have shown significant improvements in parental knowledge and adherence to safe sleep practices, reducing the incidence of SIDS and promoting safer sleep environments for infants [1]. Personalized education and support are hallmarks of home visiting programs, which address specific concerns and cultural practices influencing compliance with safe sleep recommendations [2]. Parental workshops provide interactive sessions where caregivers can engage with experts, discuss challenges, and receive practical advice on implementing safe sleep practices [4]. A previous review focused on high-risk populations, finding that traditional safer sleep messaging may be less effective for vulnerable groups, advocating for more tailored interventions that address not only knowledge but also behavioral change components such as parental motivation and environmental factors [5]. Additionally, a recent systematic review protocol [6] aims to evaluate the effectiveness of preventive parental education from pregnancy to 1 month postpartum, focusing on infant sleep patterns, parental sleep, and postpartum depression. These reviews highlight key gaps in existing strategies, particularly for high-risk groups and early intervention periods [6].

This systematic review aims to evaluate the trends, effectiveness, and impact of educational patterns and methods within home settings on infant’s sleep. By analyzing a wide range of studies, this review seeks to identify the most effective strategies for improving sleep duration, reducing night awakenings, enhancing parental satisfaction, and promoting overall infant health. Our review adds to this body of knowledge by providing a comprehensive evaluation of educational strategies across the broader population, identifying effective methods and areas for improvement in promoting safe infant sleep practices.

The focus on “home settings” in this investigation is crucial because the majority of infant sleep-related incidents, including SIDS, occur in the home, making it the most relevant environment for intervention. Home settings offer unique challenges compared to clinical environments, as caregivers are responsible for implementing safe sleep practices without direct oversight from healthcare professionals. Educational interventions within homes, such as personalized counseling, home visits, and digital health tools, are designed to address the specific socio-cultural and economic factors that influence caregiver behavior. These interventions allow for practical, sustainable improvements in infant sleep practices in real-world contexts, particularly benefiting underserved populations. By focusing on home-based care, this study fills a gap in the literature, as many previous studies have concentrated on clinical settings, offering insights into how educational strategies can be most effective where they are most needed.

## 2. Materials and Methods

### 2.1. Search Strategy

We conducted a comprehensive literature search in line with Preferred Reporting Items for Systematic reviews and Meta-Analyses (PRISMA) guidelines [7] across multiple databases, including PubMed, MEDLINE, Scopus, and the Cochrane Library. The search strategy incorporated a combination of keywords and Medical Subject Headings (MeSH) terms related to infant’s sleep, education, home care, and effectiveness. The search terms used were: “neonatal sleep”, “infant sleep”, “newborn sleep”, “parental education”, “parental training”, “parental intervention”, “home care”, “home-based care”, “domestic care”, “effectiveness”, “outcome”, “impact”, “safe sleep practices”, “SIDS prevention”, and “sleep safety”.

Boolean operators (AND, OR) were utilized to combine search terms and refine results. We applied filters to include only studies published in peer-reviewed journals published until 20 October 2024. We also hand-searched the reference lists of relevant articles to identify additional studies. A comprehensive literature search strategy is provided in Supplementary File S2. Our review protocol was registered with the International Prospective Register of Systematic Reviews (PROSPERO) under identification number CRD42024581176.

### 2.2. Inclusion and Exclusion Criteria

The inclusion criteria for this review were as follows:

- Studies published in peer-reviewed journals.
- Studies focusing on infants (aged 0–12 months).
- Interventions implemented in home settings, such as parental education, behavioral interventions, and the use of mobile health technologies.
- Measured outcomes, including sleep patterns (e.g., adherence to safe sleep practices), parental satisfaction (e.g., satisfaction with the intervention, confidence in managing sleep), and infant’s and parents’ well-being (e.g., sleep duration, depression)
- Quantitative, qualitative, and mixed-methods research designs.
- Studies available in English.

The exclusion criteria included the following:

- Studies not published in peer-reviewed journals.
- Studies conducted in clinical or institutional settings, such as hospitals or neonatal intensive care units (NICUs).
- Articles not available in English.
- Case reports, commentaries, and editorials.

### 2.3. Quality Assessment

A comprehensive quality assessment was conducted for the included studies using appropriate tools based on their respective study designs. Randomized Controlled Trials (RCTs) were assessed using the Cochrane Risk of Bias Tool (RoB 2) [8], which evaluates biases in randomization, allocation, blinding, and outcome reporting. For longitudinal and cohort studies, the National Institutes of Health (NIH) Quality Assessment Tool [9] and the Newcastle-Ottawa Scale (NOS) [10] were employed to evaluate factors such as selection bias, outcome assessment, and follow-up adequacy. Cross-sectional studies were appraised using the Appraisal Tool for Cross-Sectional Studies (AXIS) Tool [11], while quality improvement projects were assessed using the Standards for QUality Improvement Reporting Excellence (SQUIRE) Guidelines [12]. Quasi-experimental studies were evaluated using the Risk of Bias In Non-randomized Studies of Interventions (ROBINS-I) tool [13], focusing on biases arising from non-random allocation. Single-arm feasibility studies were appraised using the National Institute for Health and Care Research (NIHR) Feasibility and Pilot Study Risk of Bias Tool [14]. This systematic approach ensured that each study’s methodological quality was rigorously evaluated, providing confidence in synthesizing findings and minimizing potential biases’ impact. The results of Quality Assessment are available in Supplementary File S3.

### 2.4. Data Extraction and Synthesis

Two independent reviewers conducted data extraction using a standardized form. The data extraction form included the following elements:

- Study design (e.g., randomized controlled trial, cohort study, qualitative study).
- Sample size and characteristics (e.g., number of participants).
- Intervention details (e.g., type of intervention, duration, delivery method).
- Outcome measures (e.g., sleep patterns, parental satisfaction, infant and parental well-being).
- Key findings and conclusions.

Any discrepancies between the reviewers were resolved through discussion or consultation with a third reviewer. Extracted data were synthesized using a narrative approach, categorizing results by type of intervention (e.g., educational programs, behavioral interventions, mobile health interventions) and outcomes (e.g., sleep patterns, parental satisfaction, effects on parental and infant well-being). This allowed for a comprehensive summary of the evidence, highlighting similarities and differences across studies, identifying patterns, and drawing conclusions about the effectiveness of various interventions.

### 2.5. Study Selection Process

Identification:

- Databases searched: PubMed, MEDLINE, Scopus, Cochrane Library.
- A total of 3294 records were identified through database searches.
- Duplicate records removed: 795.
- Records removed for other reasons: 722.
- Additional records identified through hand-searching of reference lists: 5.

Screening:

- Evaluation of 1782 articles.
- Titles and abstracts screened for relevance.
- 1220 articles excluded based on title and abstract.
- 562 full-text articles assessed for eligibility.

Eligibility:

- Full-text articles reviewed against inclusion and exclusion criteria.
- 498 articles excluded (reasons: not peer-reviewed, clinical setting, not meeting outcome measures).

Included:

- 64 articles met inclusion criteria after full-text review.
- Final selection: 23 studies were included in the systematic review after detailed review and data extraction.

**Figure 1:**
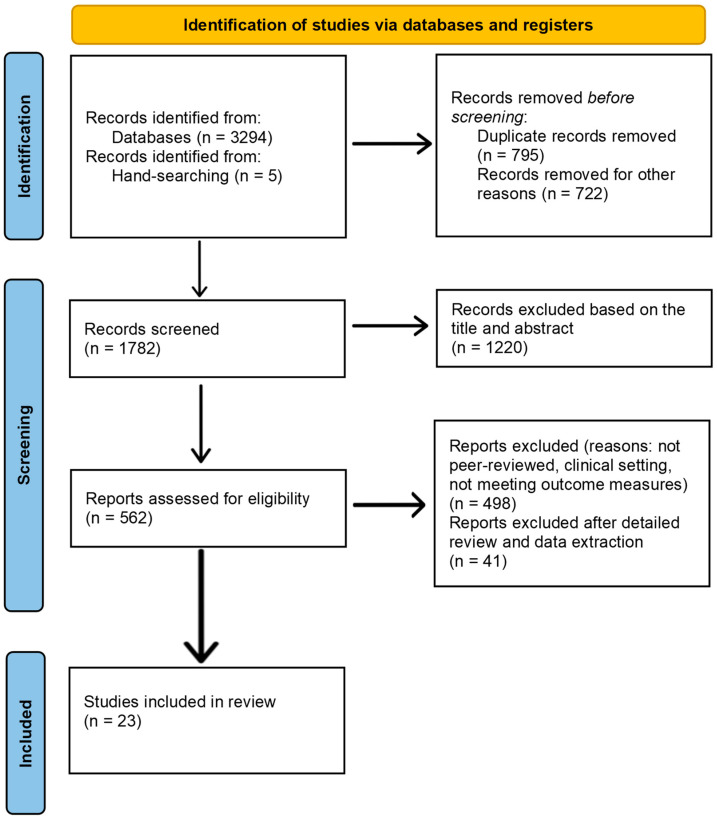
PRISMA flow diagram.

The PRISMA flow diagram for this study selection process is depicted in Figure 1.

## 3. Results

### 3.1. Included Studies

The final set of 23 studies included in the systematic review provides robust evidence to evaluate the effectiveness of home-based interventions aimed at improving infant sleep patterns, enhancing parental satisfaction, and promoting infant health.

Table 1 summarizes the studies included in the systematic review [1,2,15,16,17,18,19,20,21,22,23,24,25,26,27,28,29,30,31,32,33,34,35].

### 3.2. Trends in Educational Interventions

The review identified several trends in educational interventions for infant sleep care. Common strategies included parental education programs, digital health interventions, and personalized counseling. Hospital-based educational programs and comprehensive hospital initiatives have shown significant improvements in parental knowledge and adherence to safe sleep practices [1,17,24]. The integration of technology, such as mobile apps and online resources, has become increasingly prevalent, providing accessible and scalable solutions for parental education [22,25,26]. Moreover, home-visiting programs and community-based initiatives offer personalized education and support, addressing specific concerns and cultural practices that influence safe sleep adherence [27,29,33]. These diverse approaches highlight the importance of multifaceted interventions tailored to the unique needs of families to effectively promote safe sleep practices and improve infant health outcomes. It has been suggested that combining different educational interventions, such as hospital-based education with follow-up home visits or digital tools, leads to improved adherence to safe sleep practices and better infant health outcomes. For example, Moon et al. [24] found that combining mobile health interventions with nursing quality improvement programs resulted in significantly higher adherence to safe sleep guidelines than when these interventions were used alone. Similarly, interventions that include both in-person education and digital reminders demonstrate better parental adherence and knowledge retention compared to single-method approaches [24].

### 3.3. Effectiveness of Educational Strategies

The review of various studies highlights the effectiveness of different educational strategies in improving infants’ sleep care. These strategies have shown varying degrees of success in enhancing parental knowledge and adherence to safe sleep practices.

Several studies have demonstrated that combined educational interventions are more successful in enhancing parental knowledge and adherence to safe sleep practices. For example, hospital-based education combined with home visits or mobile health technologies have shown significant improvements in safe sleep adherence. Moon et al. [24] found that the combination of a mobile health intervention with nursing quality improvement programs led to higher adherence to safe sleep practices compared to using these interventions separately. Similarly, the study by McDonald et al. [21] highlighted the effectiveness of structured educational sessions delivered during well-child visits, which significantly improved adherence to all key safe sleep practices. These findings suggest that multifaceted approaches involving continuous reinforcement, such as combining in-person education with digital tools, are more effective than single-method interventions.

Targeted interventions and continuous follow-up have proven to be highly effective. For instance, Brashears et al. [16] enhanced screening and education by incorporating specific sleep safety questions and providing targeted education during well-child visits. This approach led to a significant reduction in unsafe sleep practices, highlighting the importance of ongoing education to ensure adherence to safe sleep guidelines. Similarly, hospital-based programs also play a crucial role in providing foundational knowledge. Goodstein et al. [1] demonstrated that a comprehensive hospital-based infant sleep safety program significantly improved parental adherence to sleep safety practices, with high retention of correct supine sleeping knowledge at follow-up, stressing the critical role of intensive, early hospital education.

In addition to in-person education, long-term parental education has shown sustained benefits in maintaining safe sleep environments. Mathews et al. [15] conducted a longitudinal cohort study, finding that continuous education reduced the use of soft bedding and improved safe sleep practices over time. This suggests that long-term parental education is key to ensuring adherence to sleep safety practices as infants grow.

Early educational interventions delivered shortly after birth further enhance knowledge and adherence. McDonald et al. [23] evaluated a randomized controlled trial where safe sleep health education was provided at a 2-week well-child visit. This early intervention significantly improved parents’ understanding and practice of safe sleep behaviors, underscoring the need to engage parents early in the postpartum period.

Mobile health interventions have emerged as effective tools in promoting safe sleep practices, particularly when combined with other strategies. Moon et al. [24] found that mobile health interventions significantly improved adherence to safe sleep guidelines, whereas a nursing quality improvement program alone did not yield the same results, highlighting the potential of digital platforms to reinforce education. Moreover, Moon et al. [25] identified that mobile health interventions influenced maternal attitudes and social norms, leading to higher adherence to safe sleep practices like supine sleep positioning and room-sharing without bed-sharing. These findings emphasize the importance of addressing not just knowledge but also attitudes and behaviors through accessible digital tools.

Finally, tailored and community-based interventions also play a crucial role. Thompson et al. [32], in the “Delta Healthy Sprouts” study, highlighted that tailored educational interventions addressing both infant sleep and activity led to improvements in maternal knowledge and adherence to sleep duration recommendations. However, some gaps, such as knowledge about tummy time, remain, indicating a need for more focused content in specific areas. Salm Ward et al. [2] evaluated a crib distribution program paired with safe sleep education, showing that providing practical tools like cribs, along with education, significantly improved adherence to safe sleep practices. A mixed methods study by Hubel et al. [34] investigating the promotion of infant safe sleep and breastfeeding at the community level revealed gaps in both promotion efforts and outcomes. The study analyzed state-level data from the Pregnancy Risk Assessment Monitoring System (PRAMS) and found that disparities in infant safe sleep practices and breastfeeding rates persist, particularly among non-Hispanic Black and American Indian/Alaskan Native populations. The qualitative component highlighted the importance of conversational approaches in promoting safe sleep and breastfeeding, with community collaboration identified as a key factor in addressing organizational capacity limitations. The study concluded that tailored program offerings and enhanced community collaboration could help reduce disparities in infant health outcomes, particularly in underserved populations. These findings underscore the potential of community-level interventions to improve both safe sleep and breastfeeding practices, though more efforts are needed to overcome barriers to access and uptake.

A quasi-experimental study [35] evaluated the effects of a structured sleep education program on mothers’ knowledge and attitudes toward infant sleep. The study included 208 mothers with infants aged 5–12 months from all Jordanian governorates. The intervention group showed a significant improvement in mothers’ sleep knowledge over time (*p* < 0.001), particularly regarding the benefits of establishing bedtime routines. However, there was no significant improvement in mothers’ attitudes toward infant sleep (*p* = 0.011), suggesting that while knowledge can be increased through education, changing attitudes may require a longer intervention period.

### 3.4. Maternal/Parental Satisfaction Outcomes

The study by Leichman et al. [21] on the Customized Sleep Profile (CSP) intervention assessed parental satisfaction with the mHealth behavioral sleep intervention delivered via a smartphone application. Caregivers reported high levels of satisfaction with the personalized recommendations and psychoeducation provided by the CSP. Specifically, 52.4% of caregivers of infants identified as problem sleepers perceived improvements in their infant’s sleep post-intervention, which contributed to the high satisfaction levels. The convenience of the app, coupled with the relevance of the tailored advice, was highlighted as a key factor in the positive feedback. Parents also appreciated the noticeable improvements in their infants’ sleep patterns, including fewer night wakings and longer sleep stretches. This, in turn, led to reduced parental stress and an overall enhancement of family well-being.

Personalized and culturally relevant approaches were particularly well-received by participants. Salm Ward et al. [33], in their evaluation of the “My Baby’s Sleep” (MBS) intervention, found that African American families from under-resourced neighborhoods expressed high levels of satisfaction with the program. The personalized nature of the intervention, along with its cultural relevance, addressed specific family needs and practices, fostering a sense of support and engagement. The structured sessions, educational materials, and guidance from supportive coaches were consistently praised, contributing to both high retention rates and a positive reception of the program.

Prenatal and postnatal guidance also played a significant role in increasing maternal confidence and satisfaction. Sweeney et al. [31] tested a behavioral–educational sleep intervention delivered to first-time mothers, and participants reported high satisfaction with both the prenatal guidance and the postnatal follow-up support. Mothers in the sleep intervention group (SIG) experienced an increase in nocturnal sleep duration and improved perceptions of their own sleep quality, which helped to reduce stress and boost confidence in managing both maternal and infant sleep. The participants found the educational content highly valuable, and their overall positive experiences contributed to higher satisfaction in their parenting roles [31].

### 3.5. Impact on Infant and Parental Well-Being

Educational interventions aimed at promoting safe sleep practices have demonstrated positive impacts on both infant and parental well-being. Improved sleep patterns in infants contribute to better health outcomes, while parents benefit from increased confidence in sleep management, reduced stress, and improved mental health.

Paul et al. [27] investigated the Responsive Parenting (RP) intervention, which included a sleep component aimed at obesity prevention. Infants in the RP group exhibited longer sleep durations, more consistent bedtime routines, and a greater ability to self-soothe compared to the control group. These improved sleep patterns were associated with broader developmental benefits, such as enhanced cognitive, psychomotor, and socioemotional development. Moreover, better sleep supported infants’ emotional regulation and behavioral management while also improving parental mental health, thereby fostering a healthier developmental environment. This intervention likely contributed to better overall health and developmental outcomes. Similarly, Santos et al. [29] found that behavioral sleep hygiene counseling significantly increased infants’ average nighttime sleep duration and had positive effects on their linear growth and neurocognitive development. This highlights the broader developmental benefits of improving infant sleep hygiene through educational interventions.

Sweeney et al. [31] emphasized the dual benefit of educational interventions for both mothers and infants. Their study demonstrated that a behavioral–educational sleep intervention significantly increased nocturnal sleep duration for mothers, enhanced their perceptions of sleep quality, and boosted their confidence in managing infant sleep. This underlines the importance of addressing parental well-being in interventions, as improved parental mental health can positively influence infant care.

Rouzafzoon et al. [28] assessed the effectiveness of a preventive behavioral sleep intervention (BSI) on infant sleep patterns and maternal health. The intervention significantly improved infant sleep patterns, including longer nighttime sleep periods and earlier bedtimes. Additionally, maternal sleep quality improved, and depression levels decreased, indicating a comprehensive benefit to both infant and family well-being. Maternal sleep quality was assessed using the Pittsburgh Sleep Quality Index (PSQI), and depression levels were measured using the Edinburgh Postnatal Depression Scale (EPDS), providing validated insights into the positive effects of the intervention on family health. In contrast, Santos et al. [30] evaluated a sleep intervention in Pelotas, Brazil, and found no statistically significant differences in nighttime sleep duration between the intervention and control groups at any age. This suggests that while some educational interventions succeed in improving sleep patterns, others may not effectively increase sleep duration, particularly in certain populations or contexts.

## 4. Discussion

This systematic review examined the effectiveness of domiciliary educational programs aimed at enhancing safe sleep practices among caregivers responsible for infants between birth and 12 months of age. Our findings highlight the positive impact of these interventions, which include hospital-based programs, home visits, and digital tools, in improving parental knowledge, adherence to safe sleep guidelines, and overall satisfaction. These strategies have been shown to reduce unsafe sleep behaviors and enhance infant health outcomes. However, our review underscores the need for future research to address the variability in intervention methods, cultural contexts, and geographic focus while also advocating for standardized outcome measures and long-term studies to fully assess the sustained impact of these interventions.

In contrast, Shiells et al. [5], in their systematic review, focus on high-risk populations, particularly families in deprived neighborhoods, whose infants face a greater risk of sudden unexpected death in infancy (SUDI). Their analysis utilizes the COM-B model and the Theoretical Domains Framework to explore the behavioral change components of safer sleep interventions. While similar to our review in terms of advocating for educational interventions, Shiells et al. [5] emphasize that traditional safer sleep messaging may not be as effective for high-risk groups. Instead, they advocate for interventions that go beyond knowledge dissemination and focus on modifying parental capability, opportunity, and especially motivation—the latter being crucial in driving safer sleep practices in vulnerable populations. Their findings suggest that practitioners need to tailor their approaches to these high-risk groups by incorporating practical demonstrations and peer support and addressing parents’ goals and emotional factors, which are often barriers to adopting safer sleep behaviors.

Our review contributes to the literature by providing a comprehensive evaluation of educational strategies in the broader population, while Shiells et al. [5] fill a critical gap by analyzing behavior change techniques in the context of SUDI and high-risk infants. The emphasis by Shiells et al. on addressing the motivational and environmental factors that influence behavior offers a more targeted approach for populations where traditional methods may fall short. Together, these reviews complement one another by providing a well-rounded understanding of how safer sleep interventions can be optimized, both for general populations and for those at heightened risk of infant mortality.

Of interest, a recently published systematic review protocol aims to evaluate the effectiveness of preventive parental education provided from pregnancy to 1 month postpartum on infant sleep, postpartum parental sleep, and parental depression [6]. Given that untreated infant sleep problems can persist into childhood and are associated with developmental issues, this review will consider experimental and quasi-experimental studies focusing on interventions that promote healthy sleep habits, such as self-soothing and independent sleeping techniques, starting during pregnancy. The review aims to assess outcomes related to infant sleep patterns, the number of parental awakenings, nocturnal sleep time, and parental depression. It also aims to compare these outcomes to standard care or alternative interventions and use meta-analysis where possible. The findings are expected to inform future recommendations for improving both infant and parental health by targeting sleep-related problems early in the perinatal period [6].

Our review reveals a consistent pattern across studies: educational interventions play a pivotal role in substantially improving parental compliance with safe sleep protocols. For example, hospital-based interventions, such as the comprehensive sleep safety programs described by Goodstein et al. and McDonald et al., provided foundational education that improved parental knowledge at the point of discharge and maintained adherence over time [1,23]. These findings underscore the value of intensive early education delivered before families transition to home care. Goodstein et al. found that 99.8% of parents were aware of the supine sleep position as the safest practice at discharge, with an 84.9% adherence rate at four months follow-up [1]. This high retention rate highlights the importance of structured and early intervention.

Several studies show that continuous or follow-up education is crucial for maintaining adherence to safe sleep guidelines. Mathews et al. emphasized the long-term benefits of ongoing education, noting a significant reduction in the use of soft bedding in infant sleep environments over time [14]. Similarly, Brashears et al. demonstrated that incorporating follow-up calls and personalized sleep education during well-child visits led to a substantial reduction in unsafe sleep practices, reinforcing the need for continuous reinforcement of safe sleep practices throughout infancy [15].

Mobile health (mHealth) interventions have emerged as effective tools for reinforcing safe sleep practices, especially in combination with other methods. Moon et al. evaluated a mobile health intervention that provided daily educational messages and videos to mothers over a 60-day period, leading to significant improvements in adherence to safe sleep practices, such as supine sleeping and room-sharing without bed-sharing [24]. Notably, combining mHealth with in-person nursing support resulted in better adherence compared to either intervention alone, suggesting that digital tools can effectively complement traditional education.

The cultural context in which these interventions are implemented plays a pivotal role in their effectiveness. Studies like those by Salm Ward et al. demonstrate that tailored educational interventions, which account for cultural practices and specific family dynamics, can enhance parental adherence to safe sleep practices in underserved communities [32]. Addressing specific cultural practices and socioeconomic factors allows for more practical and sustainable changes in behavior, particularly in high-risk populations.

The delivery method of educational interventions also influences their effectiveness. Hospital-based programs tend to provide a structured and standardized approach, as seen in Goodstein et al., but may lack the personalized follow-up found in home-based interventions [1]. Home visiting programs, as discussed by Santos et al., offer a tailored approach that accounts for individual family needs and cultural factors but may require more resources and logistical support to implement [29]. Digital interventions, as explored by Moon et al., provide scalable solutions that are accessible to a broader audience, although they may lack the personal touch and immediate feedback provided by in-person education [24].

Despite the effectiveness of these interventions, variability remains in terms of outcomes, particularly regarding the long-term sustainability of behavior change. Studies like those by Paul et al. and Santos et al. highlight the need for standardized outcome measures and extended follow-up periods to fully assess the long-term impact of educational strategies on infant sleep patterns [27,29]. Moreover, the variability in intervention delivery, as well as differences in cultural and geographic contexts, suggests that future research should focus on tailoring interventions to specific populations and settings.

The implementation of safe sleep practices for infants is influenced by a combination of caregiver knowledge, cultural factors, and the quality of information provided by healthcare professionals. Despite high levels of parental education, only a few caregivers receive safe sleep education from healthcare providers. This highlights the critical need for comprehensive and effective educational programs by healthcare providers to ensure caregivers are well-informed about safe sleep practices. Cultural practices, parental fatigue, and convenience also influence adherence, suggesting that interventions need to be culturally sensitive and address practical challenges [36].

In addition to caregiver education and healthcare-provider communication, socioeconomic and demographic factors also significantly impact the implementation of safe sleep practices. Lower socioeconomic status, lack of access to healthcare resources, and lower educational attainment among caregivers have been associated with decreased adherence to safe sleep recommendations. Additionally, racial and ethnic disparities play a role, with minority groups often facing barriers such as language differences and mistrust of healthcare systems [37].

Several barriers hinder the consistent implementation of safe sleep practices, including gaps in healthcare providers’ knowledge and communication. Angal et al. noted that although 98% of physicians in South Dakota understood the importance of discussing SIDS with parents, many did not provide comprehensive advice covering all aspects of safe sleep. Factors such as years since training significantly influenced whether providers shared detailed information on safe sleep, with those more recently trained being more likely to do so [38]. Similarly, Cole emphasized that inconsistent advice from healthcare professionals leads to confusion among caregivers, reducing adherence to safe sleep practices [39]. In another study by Dorjulus et al., barriers such as language differences, cultural beliefs, and socioeconomic factors were identified as significant obstacles to the dissemination of safe sleep information [40].

Educational interventions aimed at promoting safe sleep practices for infants have shown varying degrees of effectiveness. One successful strategy includes direct education of healthcare professionals, which subsequently influences their interactions with caregivers. For example, Moon et al. emphasized the importance of educating healthcare providers about safe sleep guidelines and addressing misconceptions that could hinder adherence [41]. This education allows providers to model safe sleep practices consistently and provide accurate information to parents. It has been shown that healthcare providers who receive comprehensive training on safe sleep guidelines are more likely to counsel parents effectively and demonstrate these practices in clinical settings, thereby improving overall adherence to safe sleep recommendations [41].

Moreover, incorporating culturally sensitive approaches and addressing specific barriers faced by different communities are essential for the success of these interventions. Naugler et al. [42] pointed out that interventions tailored to address cultural beliefs and practices surrounding infant sleep can enhance the effectiveness of educational programs. For example, community workshops and targeted messaging that respect and integrate cultural values have been shown to improve the acceptance and implementation of safe sleep practices. This approach ensures that the educational content is relevant and relatable to diverse caregiver populations, thereby increasing the likelihood of behavior change and adherence to recommended practices [42].

While this systematic review offers valuable insights into safe infant sleep practices and effective interventions, several limitations must be noted. The heterogeneity of studies, varying in population, intervention type, and outcome measures, complicates direct comparisons and generalizations. Publication bias, favoring studies with positive findings, may skew effectiveness assessments. The limited geographic scope, with a concentration in the United States and Australia, reduces the generalizability to diverse cultural and socioeconomic contexts. Short-term follow-up data restrict understanding of long-term behavioral changes, necessitating longitudinal studies. Variability in the implementation of intervention and reliance on self-reported data introduce potential biases and inconsistencies. The lack of standardized outcome measures complicates data synthesis, while small sample sizes in some studies limit statistical power and increase the risk of type II errors. Addressing these limitations in future research is essential for developing more effective, universally applicable interventions to promote safe infant sleep and reduce sleep-related infant deaths.

Future public health initiatives should enhance educational campaigns with clear, consistent messaging and culturally tailored interventions involving family members to improve adherence to safe sleep practices. Integrating user-friendly digital tools like mHealth and EHR portals is essential for broadening the reach and efficacy of educational efforts and providing personalized feedback and reminders. Special attention must be given to high-risk groups, such as young, less educated, and minority mothers, through targeted support programs like My Baby’s Sleep (MBS). Continuous professional training for healthcare providers is necessary to bridge gaps in knowledge and practice regarding safe sleep guidelines. Future research should investigate the long-term effects of these interventions on infant health, using longitudinal studies to assess behavioral sustainability. Finally, community involvement, including collaboration with local leaders and networks, is critical for effectively disseminating safe sleep messages and ensuring culturally relevant interventions.

## 5. Conclusions

This systematic review highlights home-based educational interventions’ significant impact on improving infant sleep care. These interventions effectively enhance parental knowledge and adherence to safe sleep practices, contributing to better infant health outcomes and higher parental satisfaction. The findings underscore the need for multifaceted and culturally tailored educational strategies to address the unique needs of diverse families. Future research should focus on addressing current limitations, such as study heterogeneity and short-term follow-up, to develop more effective, universally applicable interventions that promote safe infant sleep and reduce sleep-related infant mortality.

## Figures and Tables

**Table 1:** Summary of studies included.

N	Authors	Year	Study Design	Sample	Intervention	Outcome Measures	Key Findings
1	Brashears et al. [16]	2020	Quality Improvement Project (Prospective)	199 caregivers	The intervention included updating the screening forms to include four specific PRAMS questions relevant to infant sleep, followed by education tailored to each caregiver’s needs. Each family received a callback 2 weeks after the WCC to rescreen for sleep practices. This targeted educational intervention took place over the first 6 months of the infant’s life and was reinforced during multiple well-child visits. A board book about safe sleep was also provided to all participants to facilitate the education.	Identification of unsafe sleep practices; targeted education effectiveness; reduction of unsafe practices.	The project identified that **55.8%** of caregivers reported one or more unsafe sleep practices at the well-child check (WCC) using the new screening form. After targeted education was provided, a follow-up screening 2 weeks later showed a **significant reduction in unsafe sleep practices**, although the exact percentage of reduction is not provided. A chi-square test indicated a significant decrease in unsafe behaviors post-intervention (*p* = 0.046). The intervention was evaluated by screening parents using updated questions from the Pregnancy Risk Assessment Monitoring System (PRAMS), and follow-up calls were made 2 weeks after the well-child visit. The updated screening better captured actual unsafe sleeping behaviors, such as the use of blankets and sleeping in devices not intended for sleep, which enabled more targeted and effective education.
2	Canty et al. [17]	2020	Randomized Controlled Trial	109 mothers	The intervention group received personalized feedback through the patient portal based on photographs of the infant’s sleep environment submitted at 1 and 2 months. Mothers were asked to submit two photographs of their infant’s sleeping surface from different angles at 1 and 2 months postpartum. The feedback highlighted compliance with or deviations from the AAP safe sleep guidelines, including recommendations for room-sharing, supine sleep position, and removing soft objects or loose bedding from the sleeping area.	Feasibility of using patient portals for infant sleep safety; adherence to AAP safe sleep guidelines based on submitted photographs.	At the 2-month follow-up, **55.6%** of the intervention group infants who submitted photographs met all safe sleep criteria, compared to **45.4%** in the control group. However, the difference was not statistically significant (*p* = 0.75). When excluding sleep location as a criterion, **83%** of intervention group infants met all safe sleep guidelines versus **68%** in the control group (*p* = 0.46). The study assessed whether personalized feedback provided through a patient portal based on photographs of infant sleep environments could improve adherence to the American Academy of Pediatrics (AAP) safe sleep guidelines. While mothers who submitted photographs generally adhered to supine sleeping recommendations (100%), non-adherence to other safe sleep recommendations persisted. Common reasons for non-adherence included sleeping in a room without a caregiver (43%), loose bedding (15%), and other objects on the sleep surface (8%).
3	Carlin et al. [18]	2018	Randomized Controlled Trial	1194 mothers (958 completed first follow-up, 716 completed second follow-up, 637 completed all follow-ups)	Mothers in the intervention group received **enhanced messaging** emphasizing both SIDS risk reduction and suffocation prevention. Mothers in the control group received only standard messaging about safe sleep practices. Follow-ups were conducted at **2–3 weeks, 2–3 months, and 5–6 months** postpartum. However, there was **no significant impact** of the enhanced messaging in maintaining supine sleep positioning compared to the control group.	Infant sleep position at six months; maternal knowledge and attitudes; self-efficacy regarding sleep practices.	Over the first 6 months, **supine sleep positioning declined from 95.9% at 2–3 weeks to 79.9% at 5–6 months** in both the standard and enhanced messaging groups. There was no significant difference between the groups in terms of sleep positioning practices, with enhanced messaging not showing any additional effect in reducing non-supine sleep positioning. The study showed no statistically significant difference between the control and intervention groups regarding the effectiveness of messaging on changing infant sleep positions over time. **By 5–6 months, only 79.9% of infants were placed in the supine position** despite high awareness (94%) of the AAP guidelines. The messaging interventions did not significantly alter parental behavior regarding safe sleep positioning.
4	Dowling et al. [19]	2018	Longitudinal Study	15 mothers of preterm infants born before 37 weeks’ gestation	The **CaPSS** educational module was delivered over five sections, including information on safe sleep practices, protective infant care practices, and infant sleep regulation. Each section was designed to take about **10 min**, and mothers could view the sections at their own pace. The module aimed to not only inform about the recommended safe sleep practices but also provide the **rationale behind each recommendation**, explained in lay language. Mothers completed a **pre-discharge survey**, the educational module, and a **post-discharge survey** 4 weeks after leaving the hospital. The module also included additional content specific to the needs of preterm infants, addressing topics such as adjusting to life at home after discharge.	Mothers’ knowledge of SIDS Risk-Reduction Recommendations (RRRs); changes in plans for infant care post-discharge; adherence to SIDS RRRs 1-month post-discharge.	After participating in the **Caring about Preemies’ Safe Sleep (CaPSS)** educational program, the mothers’ knowledge of SIDS risk-reduction recommendations (RRRs) significantly increased. Before viewing the educational module, **40%** of the mothers felt they knew only “a little” about SIDS, and after completing the module, **100%** of mothers reported an increase in knowledge about SIDS recommendations. The increase in knowledge was statistically significant (*p* = 0.000) immediately after viewing the module and remained significant at 1 month post-discharge (*p* = 0.012). Although there was an increase in knowledge, **actual adherence to some safe sleep practices remained suboptimal** after discharge. For example, **86.7%** of mothers had planned to have their baby sleep in their room, but only **71.4%** were actually doing so 1-month post-discharge. Similarly, **46.7**% of mothers planned to breastfeed exclusively, but only **14.3%** were doing so after discharge.
5	Goodstein et al. [1]	2015	Cross-sectional Survey and Quasi-experimental Nonequivalent Control Group Study	1092 parents at hospital discharge (HD) and 490 at 4-month follow-up (F/U)	The intervention included a comprehensive ISS program that consisted of consistent modeling of safe sleep practices by all staff, mandatory viewing of the educational DVD “Safe Sleep for Your Baby: Right From the Start”, review of written materials, and a parental signature of an acknowledgment form. This intervention was delivered to all families in the postpartum units of two community hospitals. The program started at birth and continued through educational reinforcement at every interaction, ensuring that each baby had a safe sleep environment both in the hospital and at home. Parents received information primarily from nurses, with additional input from physicians and the educational DVD.	Parental knowledge of safe sleep practices; actual sleep practices at home.	At hospital discharge, 99.8% of parents were aware that the safest sleep position was supine (on the back), with 94.8% reporting their intention to always use this position. At the 4-month follow-up, 84.9% of parents continued to place their infants in the supine position (a statistically significant reduction compared to hospital discharge rates, *p* < 0.01). The effectiveness of the intervention was evaluated through surveys conducted at hospital discharge and at 4-month follow-up. A significant decline in unsafe practices was noted in various aspects of sleep safety, including room-sharing and the use of inappropriate sleep surfaces. Knowledge of the crib being the safest sleep surface remained high (99.8% at discharge and 99.5% at follow-up), but actual use of cribs in the parents’ room dropped from 91.9% at discharge to 68.2% at follow-up. The survey also revealed that retention of the recommendation for no soft objects in the crib decreased slightly from 94.7% at discharge to 85.9% in actual practice.
6	Hall et al. [20]	2015	Randomized Controlled Trial	Biological or adoptive families in the Greater Vancouver area, infants aged 5.5–8 months with moderate behavioral sleep problems (BSP)	The intervention consisted of a **two-hour group teaching session** and four **bi-weekly follow-up phone calls** over a period of two weeks. Parents were taught strategies to promote infant self-soothing and were provided with handouts, sleep–wake–feed–play charts, and controlled comforting charts to track their use of the techniques. The intervention focused on changing parental cognitions and behaviors to improve infants’ sleep through structured routines and controlled comforting techniques.	Primary: Composite measure of significant sleep disturbance (parent report and actigraphic data); Secondary: Infant’s longest sleep duration, parental sleep quality, fatigue, depression, and sleep cognitions.	The study did not focus on “unsafe sleep practices” per se, but it did measure the reduction in the severity of infants’ behavioral sleep problems. After the intervention, the proportion of infants with severe sleep problems decreased from **14% to 4%** in the intervention group, while the control group decreased from **18% to 14%**. This represents a significant relative reduction in severe sleep issues by **10%** (*p* = 0.01). The intervention was effective in reducing the number of night wakes. Parents in the intervention group reported fewer night wakings based on sleep diaries, with **31.1%** of infants in the intervention group having two or more night wakes, compared to **60.4%** in the control group (*p* < 0.001). Additionally, the longest sleep period increased significantly in the intervention group compared to the control group, with an adjusted difference of **20.02 min** (*p* = 0.05).
7	Leichman et al. [21]	2021	Real-world Study	Participants using Johnson’s Bedtime Baby Sleep app	The intervention involved the use of the **Johnson’s^®^ Bedtime^®^ Baby Sleep App**, which provided parents with a **personalized sleep profile** based on responses to the **Brief Infant Sleep Questionnaire-Revised (BISQ-R)**. The app delivered **individualized recommendations** for improving sleep, including strategies for establishing a bedtime routine, managing night wakings, and promoting self-soothing. Caregivers completed the BISQ-R at baseline and 4 to 28 days after receiving the personalized sleep recommendations. The app also provided tools for **tracking sleep** and consulting with sleep experts.	Sleep parameters; parent-perceived sleep problems.	The mHealth intervention, using the **Customized Sleep Profile (CSP)** delivered via the **Johnson’s^®^ Bedtime^®^ Baby Sleep App**, was associated with significant improvements in sleep outcomes for infants identified as problem sleepers (PSs). The **PS group** showed a **decrease in night waking frequency** from an average of **3.01 wakings** per night at baseline to **2.58 wakings** at follow-up (*p* < 0.05), with a reduction in the duration of night wakings by **17 min** on average. The **PS group** also experienced a significant increase in the **longest continuous stretch of sleep**, with an improvement of **39 min** compared to **17 min** in the non-problem sleeper (NPS) group. Total nighttime sleep increased significantly for both groups. Infants in the PS group gained an additional **50.43 min** of sleep compared to **26.90 min** in the NPS group (*p* < 0.01). The BISQ-R total score, a measure of sleep quality, improved more for the PS group compared to the NPS group, showing a greater magnitude of change in sleep quality.
8	Martins et al. [22]	2018	Longitudinal Study	200 mothers	The intervention consisted of a **15-min individual education session** on the second postnatal day, followed by the distribution of a written leaflet summarizing key points. The content focused on **normal infant sleep cycles**, the importance of establishing **sleep routines**, and strategies to promote **self-soothing** (such as putting the infant to bed while sleepy but still awake). Follow-up questionnaires were conducted at **1, 2, 4, and 6 months**.	Knowledge, attitudes, and practices regarding safe sleep.	The study focused on promoting healthy sleep habits through maternal education but did not explicitly report on reductions in unsafe practices. However, it demonstrated significant improvements in infant sleep autonomy. For instance, at 6 months of age, infants in the intervention group were **6.1 times more likely** to fall asleep in their own beds (**ORadj, 6.1; 95% CI, 3.5–10.6**) compared to the control group and **4.29 times more likely** to fall asleep alone (**ORadj, 4.29; 95% CI, 2.4–7.6**). The intervention significantly improved the development of autonomous sleep habits. By 6 months, infants in the intervention group were more likely to sleep in their own beds and fall asleep alone. Specifically, at 6 months, infants in the intervention group needed less breast or bottle feeding to fall asleep (**ORadj, 2.68; 95% CI, 1.5–4.6**) and were more likely to go back to sleep after night awakenings without parental assistance (**ORadj, 3.88; 95% CI, 2–7.5**).
9	Mathews et al. [15]	2018	Longitudinal Study	637 infants	The intervention consisted of enhanced health messaging provided to African American mothers during their hospital stay. The messaging included both verbal and written materials emphasizing the importance of following AAP guidelines to prevent both SIDS and suffocation. Mothers received follow-up telephone interviews at **2–3 weeks, 2–3 months, and 5–6 months postpartum**. The enhanced group was more likely to reduce the use of soft bedding in the infant sleep environment.	Use of soft bedding; safe sleep practices.	In the group that received enhanced messaging, the use of soft bedding decreased significantly compared to the standard messaging group. By the final follow-up at 5–6 months, **43.0%** of the enhanced messaging group used soft bedding the previous night, compared to **52.4%** in the standard group (*p* = 0.02). Additionally, the use of soft bedding in the past week was **49.2%** in the enhanced group, compared to **59.6%** in the standard group (*p* = 0.01). The enhanced messaging, which emphasized both SIDS risk reduction and suffocation prevention, led to a **26% reduction in soft bedding use the previous night** and a **30% reduction in soft bedding use during the past week** in the enhanced group compared to the standard group. These reductions were significant and suggested that the enhanced messaging was more effective at changing behavior than the standard messaging focused only on SIDS risk.
10	McDonald et al. [23]	2017	Randomized Controlled Trial	235 participants (parents/guardians)	The Safe Start intervention included a structured educational session delivered by a health educator during the infant’s **2-week well-child visit**. The session emphasized four key safe sleep recommendations: baby sleeping **alone**, on the **back**, in a **crib**, and in a **smoke-free environment**. Parents were also provided with a **sleep sack** and a **portable crib** to promote adherence to safe sleep guidelines. Follow-up home visits were conducted at **2–4 weeks and 2–3 months** to assess continued compliance with the safe sleep recommendations.	Safe sleep behaviors, knowledge, beliefs, intentions, skills, and practices related to safe sleep.	The Safe Start intervention, which included safe sleep education delivered at pediatric well-child visits (WCVs), significantly impacted parental behavior. Parents in the intervention group reported greater adherence to safe sleep practices, including placing their baby to sleep **alone** (no other people or objects), on the **back**, and in a **crib** or other safe sleep space. In total, **88%** of the parents in the intervention group followed all four recommended practices, compared to **65%** in the standard care group at the first follow-up (*p* < 0.001). The intervention was designed to increase parental self-efficacy and belief in the effectiveness of safe sleep practices. The educational session improved parents’ knowledge, intentions, and reported behaviors regarding safe sleep. The intervention group showed significantly higher compliance with safe sleep practices than the control group, especially with regard to **crib use and sleeping without soft objects**.
11	Moon et al. [24]	2017	Randomized Controlled Trial	1600 mothers of healthy term neonates	The study employed a 60-day mobile health program that included **daily text or email messages** with educational content on safe sleep practices, including short videos addressing key safe sleep recommendations. Messages were delivered more frequently in the first 11 days and then every 3–4 days for the remainder of the 60 days. The program targeted adherence to **four key practices**: supine sleep position, room-sharing without bed-sharing, avoiding soft bedding, and pacifier use.	Maternal adherence to safe sleep practices.	The mobile health (mHealth) intervention was effective in improving safe sleep practices. Mothers in the mHealth group were more likely to report placing their infants in the **supine position** (89.1% vs. 80.2% in the control group; adjusted risk difference, 8.9% [95% CI, 5.3–11.7%]), practicing **room-sharing without bed-sharing** (82.8% vs. 70.4%; adjusted risk difference, 12.4% [95% CI, 9.3–15.1%]), and avoiding **soft bedding** (79.4% vs. 67.6%; adjusted risk difference, 11.8% [95% CI, 8.1–15.2%]). **Pacifier use** was also more common in the mHealth group (68.5% vs. 59.8%; adjusted risk difference, 8.7% [95% CI, 3.9–13.1%]). While the nursing quality improvement (NQI) intervention alone did not show significant improvements in adherence to safe sleep practices, the mHealth intervention was associated with statistically significant improvements in adherence to all safe sleep recommendations. In particular, the combination of the mHealth and NQI interventions resulted in the highest adherence to **supine sleep positioning**, with **92.5%** of mothers in this group placing their infants on their backs for sleep.
12	Moon et al. [25]	2019	Randomized Controlled Trial	1263 mothers	The **TodaysBaby** mHealth intervention delivered educational videos via text or email over a **60-day period**. The videos addressed key safe sleep practices, including **supine sleep positioning**, **room-sharing without bed-sharing**, and avoiding **soft bedding**. The intervention also used the **theory of planned behavior (TPB)** to influence maternal attitudes, perceived social norms, and control over safe sleep decisions.	Infant sleep position and location.	The mobile health (mHealth) intervention significantly improved adherence to safe sleep practices. Mothers who received safe sleep (SS) videos through the mHealth intervention were more likely to place their infants in the **supine position** (adjusted odds ratio [aOR] = 1.99; 95% CI 1.43 to 2.79) and were more likely to practice **room-sharing without bed-sharing** (aOR = 2.05; 95% CI 1.65 to 2.54) compared to the control group. The mHealth intervention led to an **8.9% increase** in supine sleep positioning (from 80.2% to 89.1%) and a **12.4% increase** in room-sharing without bed-sharing (from 70.4% to 82.8%). Both attitudes and perceived social norms regarding infant sleep practices were significantly improved through the intervention, which was shown to mediate the positive effects on behavior. Specifically, **positive attitudes toward supine sleep** (aOR = 8.25; 95% CI 4.72 to 14.43) and **positive social norms for room-sharing** (aOR = 7.14; 95% CI 5.35 to 9.53) were strongly associated with adherence to safe sleep practices.
13	Nabaweesi et al. [26]	2020	Randomized Controlled Trial	16 mothers	The intervention involved training mothers to take and submit smartphone photographs of their infant’s sleep environment. These photographs were assessed for compliance with safe sleep recommendations across five key domains: sleep location, sleep surface, sleep position, presence of soft items, and hazards near the sleep area. Mothers received guidance during a **90-min home visit**, and follow-up photographs were submitted **2–4 weeks** after the visit.	Sleep safety inter-rater reliability (IRR); correlation between sleep safety assessments via photography, observation, and parental reports.	The study used smartphone technology to assess safe sleep practices, comparing parental reports and observations. A total of **76.5%** of parents were observed placing their infants in the supine position, and **82.4%** of the photographs demonstrated supine sleep position. Additionally, **94.1%** of infants were observed sleeping in their parents’ room, while **82.4%** of photographs showed the same. The study demonstrated that using smartphone photographs to assess sleep safety is a reliable and feasible method. There was **perfect agreement** (Kappa = 1.00) between expert coders for sleep position and soft items in the sleeping area without the baby present, showing that this method can effectively identify safe sleep environments. The study found that using photographs resulted in reliable assessments of sleep position and location compared to parental reporting and direct observation.
14	Paul et al. [27]	2016	Randomized Controlled Trial	279 mother–infant dyads	The **Responsive Parenting (RP)** intervention focused on establishing **consistent bedtime routines**, encouraging **self-soothing**, and promoting **early bedtimes**. Intervention content was delivered through **home visits** at **3–4, 16, 28, and 40 weeks**, with personalized sleep profiles provided at 16 and 40 weeks based on the **Brief Infant Sleep Questionnaire (BISQ)**. Sleep-related guidance included avoiding feeding just before sleep, transitioning infants to their own room by 3 months, and encouraging parents to allow time for infants to self-soothe when waking at night.	Bedtime routines, sleep location, sleep behaviors, and sleep duration.	Infants in the Responsive Parenting (RP) intervention group were more likely to adopt healthier sleep-related behaviors. For instance, at **16 weeks**, **46%** of RP infants went to bed by 8 PM compared to **24%** of the control group, and by **40 week**s, this increased to **66%** of RP infants compared to **47%** in the control group (*p* < 0.001). RP infants were also more likely to fall asleep alone in their cribs (self-soothing) at 16 and 40 weeks (44% vs. 28% at 16 weeks, *p* = 0.009; 59% vs. 46% at 40 weeks, *p* = 0.04). The intervention was effective in extending nighttime sleep duration. At **8 weeks**, RP infants slept **35 min** **longer** on average than controls (*p* < 0.001). At **16 weeks**, the RP group had **25 min** longer nighttime sleep (*p* = 0.01), and at **40 weeks**, they slept **22 min** longer than control infants (*p* = 0.01).
15	Rouzafzoon et al. [28]	2021	Randomized Controlled Trial	82 mothers and infants aged 2–4 months	The intervention group received a **90-min in-person educational session**, a booklet, and follow-up support through **weekly phone calls, text, and voice messages** over the course of 8 weeks. The session covered key strategies for improving infant sleep, such as distinguishing between day and night, creating a consistent bedtime routine, using behavioral sleep techniques like “dream feeding” and putting infants to bed drowsy but awake.	Infant sleep patterns, maternal sleep quality, and depression.	The behavioral sleep intervention (BSI) led to significant improvements in infant sleep duration. At the 8-week follow-up, the mean **nighttime sleep duration** for infants in the intervention group increased to **8.89 h** (compared to 7.53 h in the control group; *p* < 0.001). Additionally, the **longest self-regulated sleep period** (the longest period in which infants could return to sleep without signaling their mothers) increased by **1.35 h** in the intervention group compared to the control group (*p* < 0.001). The intervention was also effective in improving infant bedtime routines. The mean **bedtime** was reduced by 2 h and 40 min, shifting from **01:00 AM to 22:20 PM** in the intervention group. This change was statistically significant when compared to the control group, which showed a smaller shift from 00:52 AM to 00:25 AM (*p* < 0.001).
16	Salm Ward et al. [2]	2018	Prospective Cohort Study	132 participants	The program consisted of a **15- to 20-min group-based educational session**, covering key points of the **“Safe to Sleep”** public education campaign. Educational materials included a flipchart with talking points and illustrations on **safe sleep position (back sleeping), sleep surface (crib), and removing soft items** from the sleep environment. Participants received a **portable crib** after completing the session and had access to **follow-up support** via phone surveys conducted approximately 10 weeks after the program.	Knowledge and practices pre- and post-intervention.	After the crib distribution and safe sleep education program, there was a significant improvement in safe sleep practices. The percentage of participants placing their infants in the **supine position** increased from **62.6% at pre-test to 96.2% at post-test** (*p* < 0.001), and the percentage of participants using a crib or other recommended sleep surface increased from **59.8% to 72.7%** (*p* = 0.002). Similarly, the proportion of participants reporting no soft items in the sleep environment increased from **12.2% to 32.1%** between pre- and post-test (*p* < 0.001). The program’s educational component significantly improved parents’ knowledge of safe sleep recommendations, with knowledge retention maintained over a **10-week follow-up**. For example, knowledge that pacifier use reduces SIDS risk increased from **9.6% at pre-test to 62.4% at post-test** (*p* < 0.001). Participants demonstrated continued adherence to safe sleep recommendations at follow-up, though a slight decline in crib use and exclusive room-sharing was noted.
17	Santos et al. [29]	2016	Randomized Controlled trial	552 infants aged 3 months, born healthy, and sleeping < 15 h per 24 h and their caregivers (primarily mothers) from Pelotas 2015 Birth Cohort, Southern Brazil	The intervention consisted of a **home visit** by trained fieldworkers, where mothers received **standardized sleep hygiene counseling**. The counseling session included information on the importance of sleep for infant development, practices to promote self-regulated sleep (such as putting the baby to bed while drowsy but awake), and environmental recommendations (like reducing noise and light around bedtime). A **booklet** with intervention content was provided to the mothers, and reinforcement calls were made on the two days following the home visit, along with a reinforcement visit on the third day.	Primary: Nighttime self-regulated sleep duration at ages 6, 12, and 24 months; Secondary: Linear growth and neurocognitive development at ages 12 and 24 months.	The behavioral intervention on infant sleep hygiene aimed to promote healthier sleep patterns. The primary outcome was the **increase in nighttime self-regulated sleep**. At the **6-month follow-up**, infants in the intervention group showed an average increase of **30 min in nighttime sleep duration** compared to the control group (baseline data not yet reported). The intervention’s effect will be further evaluated at 12 and 24 months. The intervention focused on improving both the duration and quality of sleep by promoting sleep self-regulation through standardized advice given to mothers. Early results suggest a **positive impact** on sleep consolidation, but further data from 12- and 24-month follow-ups will help solidify the long-term effects on infant sleep quality and developmental outcomes.
18	Santos et al. [30]	2019	Randomized Controlled trial	586 infants and their caregivers from the Pelotas 2015 Birth Cohort, Southern Brazil	Information on sleep characteristics, improvements in the environment, the establishment of a nighttime sleep routine, and waiting before attending nocturnal awakenings was delivered to mothers in the intervention group by trained home visitors at baseline. The intervention group received a telephone call on the first and second day after the intervention and a home visit on the third day after the intervention. The intervention’s content was reinforced at health care visits for ages 6 months and 12 months. Mothers allocated to the control group were counseled on the benefits of breastfeeding for the mother’s and child’s health and given written material with content on breastfeeding.	Nighttime sleep duration was measured by interview and actigraphy at baseline and ages 6, 12, and 24 months and diaries at baseline and age 6 months. At ages 3 and 6 months, nighttime sleep self-regulation was calculated by subtracting nighttime sleep duration recorded by actigraphy from nighttime sleep duration recorded in the diaries, and at ages 12 and 24 months by subtracting nighttime sleep duration recorded by actigraphy from nighttime sleep duration obtained by interview.	At the **6-month follow-up**, infants in the intervention group had a mean nighttime sleep duration of **9.80 h** compared to **9.49 h** in the control group, a difference of **19 min** longer for the intervention group (*p* = 0.10). However, by **12 months**, the difference in nighttime sleep duration between the intervention and control groups was only **5 min** shorter for the intervention group. At **24 months**, there were no significant differences in sleep duration between the two groups. The intervention was designed to promote sleep hygiene practices through counseling. By the **6-month** follow-up, a **24.1%** adherence rate was observed in the intervention group regarding waiting **1–2 min before attending nocturnal awakenings**, compared to only **2.8%** in the control group. Additionally, **49.1%** of mothers in the intervention group placed their infants in the supine sleep position compared to **42.1%** in the control group.
19	Sweeney et al. [31]	2020	Randomized Controlled Trial	40 first-time mothers and their infants	The intervention consisted of a **2-h prenatal educational session** focusing on **normal infant sleep development**, maternal sleep stages, and strategies to promote sleep for both mothers and infants. Follow-up support was provided via **weekly phone calls for 5 weeks postpartum**, where mothers could ask questions about their own or their infant’s sleep. A booklet and relaxation audio recording were also provided as resources for the mothers.	Maternal and infant sleep duration and quality.	The intervention group (SIG) mothers experienced a significant increase in **nocturnal sleep duration by 47 min** over the second 6 weeks postpartum, while the control group (CG) showed no significant change (*p* < 0.001). Although both groups saw an increase in the longest nocturnal sleep episode (by 48 min), there were no significant differences between groups in terms of infant sleep consolidation. The intervention resulted in mothers in the SIG group reporting greater **confidence in managing infant sleep**, particularly in recognizing **tired cues** (*p* = 0.03). Mothers in the intervention group also reported an improvement in their self-reported **good night’s sleep (GNS)**, with the number of nights they slept well increasing to **3.9 nights per week** at 12 weeks postpartum compared to **2.9 nights per week** in the control group, though this difference did not reach statistical significance.
20	Thompson et al. [32]	2018	Randomized Controlled Trial	82 pregnant women (43 in control group and 39 in experimental group)	The intervention was delivered through **monthly home visits**, where Parent Educators discussed key elements of infant activity and sleep, including back-to-sleep positioning and sleep duration recommendations. The intervention also included follow-up support during **27 group meetings** where sleep recommendations were reinforced. Safe sleep recommendations, such as the **Safe to Sleep^®^** campaign, were covered during three of these group meetings.	Maternal gestational weight gain, postpartum weight control, childhood obesity prevention, infant activity, and sleep behaviors.	Knowledge about infant sleep position improved significantly. At baseline, **70.7%** of mothers knew that placing a baby to sleep on their back is the safest practice. By the study’s end, this increased to **97.8%** (*p* < 0.001). However, compliance with the back-to-sleep recommendation was suboptimal. Only **20%** of mothers consistently adhered to the recommendation for the full 12 months, with a median time to non-compliance of **7.8 months**. The intervention, part of the Delta Healthy Sprouts program, included educational components focusing on infant sleep practices, such as supine positioning, sleep duration, and establishing regular bedtimes. Despite the improvement in knowledge, adherence to the back-to-sleep recommendation declined over time, indicating the need for additional reinforcement of safe sleep practices beyond the initial education.
21	Salm Ward et al. [33]	2021	Single-arm Feasibility and Acceptability Study	17 participants (eight mothers, nine co-caregivers)	The MBS intervention included **four in-home sessions** spaced over **7 months**, starting during the last trimester of pregnancy and continuing until the infant was about 4 months old. The sessions covered topics such as planning the infant’s sleep environment, co-caregiver involvement, and addressing common challenges like infant fussiness and sleep transitions. Families also received a **travel bassinet** and **safe sleep board book** to facilitate the intervention.	Feasibility, acceptability, maternal self-efficacy, support, knowledge, attitudes, and sleep practices.	**My Baby’s Sleep (MBS)** intervention was found to be feasible for the population, with eight **African American families** completing the study. Nearly all of the sessions were delivered as planned, though **46.9% of visits** occurred outside the scheduled timeframe due to family scheduling conflicts. **Acceptability of the Intervention:** The intervention had high acceptability, as evidenced by **qualitative feedback** from participants and mean evaluation scores. Mothers rated the intervention sessions as highly helpful, with responses such as: “**Today’s visit was helpful**” rated **3.0 out of 3** across all sessions. Co-caregivers also expressed positive feedback, stating they learned critical information about safe sleep practices. Additionally, **mothers and co-caregivers** reported an increase in their understanding of safe sleep practices, such as the importance of **supine sleeping and removing suffocation hazards** from the infant’s sleep area. Families particularly appreciated the **interactive materials**, such as the Safe to Sleep^®^ videos and card-sorting activities.
22	Huber et al. [34]	2024	Mixed Methods	Seven key informant interviews; PRAMS and OPAS data from several states	Community-level ISS and breastfeeding promotion using conversational approaches, peer counseling, home visits, and risk-mitigation strategies. Focus on marginalized groups (Black, AIAN, rural populations).	Gaps between promotion and adherence to safe sleep practices; high recommendations, but lower parental adherence to safe sleep behaviors. Persistent racial/ethnic disparities (AIAN, Black, API groups showing lowest outcomes) and geographic disparities (urban vs. rural).	The intervention showed partial effectiveness. Although high provider recommendation rates were achieved (92–97%), actual adherence to safe sleep practices was lower (76% of parents reported placing infants to sleep alone). Disparities remained based on race/ethnicity and geography. Conversational approaches may help improve outcomes, but capacity issues and cultural barriers limit full effectiveness.
23	Abuhammad et al. [35]	2024	Quasi-experimental (non-equivalent group design)	208 mothers (97 intervention, 111 control) with infants aged 5–12 months	The intervention was a structured educational program aimed at improving mothers’ knowledge and attitudes toward infant sleep. The program provided the following: Information on infant sleep patterns, sleep regulation, and common sleep challenges. Advice on managing poor sleep habits like late bedtime, sleep resistance, and fragmented sleep. Explanation of sleep regulation mechanisms, such as circadian rhythms, melatonin, and sleep pressure. The program was delivered via both online platforms (WhatsApp, Telegram, Messenger) and in-person sessions using PowerPoint presentations in both Arabic and English. The duration was around 5 h, with flexibility to fit participants’ schedules.	Mothers’ knowledge and attitudes about infant sleep, measured pre- and post-intervention.	Mothers in the intervention group showed a significant improvement in knowledge about infant sleep after the educational program. Knowledge scores improved significantly from baseline to follow-up (B = 0.236, *p* < 0.001). In contrast, the control group showed no significant improvement in knowledge over time. **Impact on Attitudes:** There was no significant positive impact on mothers’ attitudes towards infant sleep in the intervention group over time (*p* = 0.011). While there was some improvement in attitude scores, these changes were not statistically significant compared to the control group. The attitudes measured included mothers’ beliefs about their infants getting enough sleep, having healthy sleep habits, and their willingness to change their infant’s sleep patterns or consult a doctor about sleep issues. There was a positive correlation between mothers’ knowledge and attitudes toward infant sleep. Mothers with higher knowledge scores also tended to have higher attitude scores (B = 0.018, *p* < 0.001). The program was highly effective in improving mothers’ knowledge about infant sleep. The significant improvements in knowledge suggest that the educational content was well-structured and comprehensible. The program was less effective in changing attitudes. While knowledge increased significantly, mothers’ attitudes toward their infant’s sleep did not change as expected. This may indicate that changing attitudes requires more time or a different approach beyond just knowledge dissemination. The flexibility of the program’s delivery (online and in-person options) was a strength, allowing more mothers to participate. However, more engagement may be necessary to shift deeply ingrained attitudes toward infant care.

AAP: American Association of Pediatrics; BSI: behavioral sleep intervention; BSP: behavioral sleep problems; MBS: My Baby’s Sleep; PRAM: Pregnancy Risk Assessment Monitoring System; SIDS: sudden infant death syndrome; RRRs: Risk-Reduction Recommendations; RP: Responsive Parenting; PAT: Parents as Teachers curriculum; PATE: enhanced PAT curriculum Proof of Theorem 1; **ISS**: infant safe sleep; **PRAMS**: Pregnancy Risk Assessment Monitoring System; **OPAS**: Ohio Pregnancy Assessment Survey; **AIAN**: American Indian/Alaskan Native; **API**: Asian/Pacific Islander.

## Data Availability

The data presented in this study are available within the articles.

## Supplementary Materials

The following supporting information can be downloaded at: https://www.mdpi.com/article/10.3390/children11111337/s1, Supplementary File S2: Search Strategy; Supplementary File S3: Quality Assessment.
