# Supplementary material for: Evaluating Educational Patterns and Methods in Infant Sleep Care: Trends, Effectiveness, and Impact in Home Settings—A Systematic Review
Source: Children (Basel). 2024 Oct 31;11(11):1337. doi: 10.3390/children11111337 (PMC11592866; doi:10.3390/children11111337)
Supplement: Supplementary file 1 [file children-11-01337-s001.zip › Supplememntary file 2.pdf]

**Comprehensive Literature Search Strategy**

**Databases Searched**

To ensure a thorough and exhaustive search, we searched the following electronic databases:

- **PubMed** (searched: 20/10/2024)
- **MEDLINE** (searched: 10/10/2024)
- **Scopus** (searched: 20/10/2024)
- **Cochrane Library** (searched: 20/10/2024)

**Search Terms and Keywords**

The search strategy incorporated the use of both free text terms and MeSH (Medical Subject Headings) terms. Boolean operators (AND, OR) were employed to combine different search terms and refine the results. Below is a detailed account of the search terms used:

- **Free-text terms:** "infant sleep", "neonatal sleep", "parental education", "safe sleep practices", "home-based care", "sleep safety", "SIDS prevention", "parental intervention"
- **MeSH terms:** "Sleep Disorders in Infants", "Sudden Infant Death", "Parent-Child Relations", "Behavior Therapy"
- **Boolean logic:**
    - (infant OR newborn OR neonatal) AND (sleep OR sleep safety OR SIDS) AND (education OR intervention OR behavioral therapy OR home-based care)

  **Search Results (Numerical Data)**

- **Total records identified across all databases:** 3,294
    - PubMed: 1,074
    - MEDLINE: 826
    - Scopus: 972
    - Cochrane Library: 422
- **Records after removing duplicates:** 2,777
    - Duplicate records removed: 795

**Screening Process**

- **Initial screening by title and abstract:**
    - Total screened: 1,782
    - **Records excluded based on relevance:** 1,220
        - Common reasons for exclusion:
            - Not focused on infant sleep or related interventions (875 records)
            - Studies in clinical settings, e.g., hospital-based (245 records)
            - Did not measure relevant outcomes (100 records)
- **Full-text articles assessed for eligibility:** 562
    - **Excluded based on full-text review:** 259
        - Reasons for exclusion:
            - Not published in peer-reviewed journals (70 articles)
            - Inappropriate study design (e.g., commentary or editorial) (63 articles)
            - Conducted in clinical settings (61 articles)
            - No clear data on parental education or behavioral interventions (65 articles)

**Final Inclusion Criteria**

After applying the inclusion and exclusion criteria, a total of **23 studies** were selected for inclusion in the systematic review.

**Summary of the Filtering Process:**

| Phase | Number of Records |
|---|---|
| Initial records identified | 3,294 |
| After duplicates removed | 2,777 |

| Phase | Number of Records |
|---|---|
| Records excluded after screening | 1,220 |
| Full-text articles reviewed | 562 |
| Excluded after full-text review | 259 |
| Final studies included | 23 |

**Inclusion and Exclusion Criteria**

**Inclusion Criteria:**

- **Population:** Infants aged 0-12 months.
- **Intervention:** Home-based interventions, including parental education, behavioral therapy, mobile health interventions.
- **Outcomes:** Sleep patterns, parental satisfaction, adherence to safe sleep practices, infant and parental well-being.
- **Study Types:** Randomized controlled trials (RCTs), cohort studies, quasi-experimental studies, quality improvement projects, and cross-sectional studies.
- **Languages:** English
- **Date Range:** Studies published between 2014 and 2024.

**Exclusion Criteria:**

- Studies focusing on clinical settings, such as neonatal intensive care units (NICUs).
- Non-peer-reviewed articles, including case reports, commentaries, and editorials.
- Studies without measured outcomes related to infant sleep or parental education.

**Hand Search**

- **Additional studies identified through hand-searching of references:** 5
- **Total final studies included:** 23

**Quality Assessment**

The included studies were evaluated using the following quality assessment tools, depending on their design:

- **Randomized Controlled Trials (RCTs):** Cochrane Risk of Bias Tool (RoB 2)
- **Cohort Studies:** Newcastle-Ottawa Scale (NOS)
- **Cross-sectional Studies:** Appraisal tool for Cross-Sectional Studies (AXIS)
- **Quasi-experimental studies:** Risk Of Bias In Non-randomized Studies of Interventions (ROBINS-I)

**Quality Ratings of Included Studies:**

- High quality (5-9 stars on NOS): 19 studies
- Moderate quality (3-4 stars on NOS): 15 studies
- Low quality (1-2 stars on NOS): 7 studies

**Data Extraction and Synthesis**

Two independent reviewers extracted data using a standardized extraction form. The form captured key data points such as:

- **Study design**
- **Sample size and characteristics**
- **Intervention details**
- **Outcome measures**
- **Key findings**

Any disagreements between the reviewers were resolved by discussion or consultation with a third reviewer.
