# Supplementary material for: Evaluating Educational Patterns and Methods in Infant Sleep Care: Trends, Effectiveness, and Impact in Home Settings—A Systematic Review
Source: Children (Basel). 2024 Oct 31;11(11):1337. doi: 10.3390/children11111337 (PMC11592866; doi:10.3390/children11111337)
Supplement: Supplementary file 1 [file children-11-01337-s001.zip › Supplemetary File 3.pdf]

Quality Assessment Results

1. Canty et al. (2020) - *Randomized Controlled Trial (RCT)*

- **Tool**: Cochrane Risk of Bias Tool (RoB 2)
- **Assessment**:
  - **Randomization Process**: Adequately reported. Mothers were randomized using patient portals.
  - **Deviations from Intended Interventions**: Some barriers were encountered in participation, with only 38% of intervention group participants submitting photographs. However, the intervention was followed as intended for those who participated.
  - **Missing Outcome Data**: Low risk. Data from both the intervention and control groups were reported, although the response rates varied between groups.
  - **Measurement of Outcome**: Objective measures (photographs) were used to assess safe sleep practices.
  - **Selective Reporting**: No evidence of selective reporting. All pre-specified outcomes were reported.
- **Overall**: The study presents low risk of bias but highlights some challenges in participation, particularly related to adherence to the intervention.

2. Carlin et al. (2018) - *Randomized Controlled Trial (RCT)*

- **Tool**: Cochrane Risk of Bias Tool (RoB 2)
- **Assessment**:
  - **Randomization Process**: Randomization was clearly described with a large sample size.
  - **Deviations from Intended Interventions**: No significant issues reported. Both control and intervention groups received standard and enhanced messages, respectively.

    - **Missing Outcome Data**: A significant number of participants were lost to follow-up (53.4% completed all interviews), increasing the risk of bias due to attrition.
    - **Measurement of Outcome**: Outcomes were assessed using validated surveys and interviews, but there was a potential for bias in self-reported practices.
    - **Selective Reporting**: All pre-specified outcomes were reported.
- **Overall**: Moderate risk of bias, primarily due to attrition and reliance on self-reported data.

3. Hall et al. (2015) - ***Randomized Controlled Trial (RCT)***

- **Tool**: Cochrane Risk of Bias Tool (RoB 2)
- **Assessment**:
    - **Randomization Process**: Proper randomization procedures were reported, and baseline characteristics were similar between groups.
    - **Deviations from Intended Interventions**: No major deviations. The study followed a robust intervention protocol with teaching sessions and follow-up calls.
    - **Missing Outcome Data**: Minimal loss to follow-up, with 96.9% of intervention and 97.9% of control group infants completing the trial.
    - **Measurement of Outcome**: Objective measures were used (actigraphy, diaries), minimizing bias in outcome assessment.
    - **Selective Reporting**: No evidence of selective reporting.
- **Overall**: Low risk of bias. The use of objective measures (actigraphy) strengthens the study's validity.

4. McDonald et al. (2017) - ***Randomized Controlled Trial (RCT)***

- **Tool**: Cochrane Risk of Bias Tool (RoB 2)
- **Assessment**:
    - **Randomization Process**: Adequately randomized with a detailed description of the process.

  - **Deviations from Intended Interventions**: Intervention was consistently delivered, but differences in counseling by pediatricians could introduce variability.
  - **Missing Outcome Data**: Follow-up rates were not clearly reported, but the home visits and surveys were likely well-executed.
  - **Measurement of Outcome**: Direct observations of sleep environments added robustness to the data.
  - **Selective Reporting**: No selective reporting detected.
- **Overall**: Low risk of bias, with a well-executed intervention and reliable outcome measures.

5. Moon et al. (2019) - ***Randomized Controlled Trial (RCT)***

- **Tool**: Cochrane Risk of Bias Tool (RoB 2)
- **Assessment**:
  - **Randomization Process**: Randomization of 1,263 mothers into intervention and control groups was adequately described.
  - **Deviations from Intended Interventions**: The mHealth intervention was delivered as planned. No significant deviations were noted.
  - **Missing Outcome Data**: Low risk of bias due to missing data, with a high number of participants completing the study.
  - **Measurement of Outcome**: Outcomes were assessed using standardized questionnaires, but there is some potential for reporting bias.
  - **Selective Reporting**: No evidence of selective reporting.
- **Overall**: Low risk of bias, particularly with strong adherence to the intervention.

6. Rouzafzoon et al. (2021) - ***Randomized Controlled Trial (RCT)***

- **Tool**: Cochrane Risk of Bias Tool (RoB 2)
- **Assessment**:
  - **Randomization Process**: Clearly described randomization process. Sample size was small but randomized appropriately.

- **Deviations from Intended Interventions**: The intervention was delivered as planned, but individual responses to sleep training may vary.
- **Missing Outcome Data**: Low attrition, with most mothers completing the post-intervention assessments.
- **Measurement of Outcome**: Objective measures such as sleep diaries and depression scales were used, minimizing bias.
- **Selective Reporting**: All planned outcomes were reported.

- **Overall**: Low risk of bias, with consistent and well-documented delivery of the intervention.

7. Santos et al. (2016) - ***Randomized Controlled Trial (RCT)***

- **Tool**: Cochrane Risk of Bias Tool (RoB 2)
- **Assessment**:
  - **Randomization Process**: Adequate randomization with detailed methodology.
  - **Deviations from Intended Interventions**: The intervention was conducted consistently, with reinforcement visits to ensure adherence.
  - **Missing Outcome Data**: Low risk, as follow-up data were collected for most participants.
  - **Measurement of Outcome**: Objective measures such as actigraphy were used to assess sleep duration.
  - **Selective Reporting**: All pre-specified outcomes were reported.
- **Overall**: Low risk of bias, with robust and objective measurement of outcomes.

8. Salm Ward et al. (2021) - ***Single-arm Feasibility Study***

- **Tool**: National Institute for Health and Care Research (NIHR) Feasibility and Pilot Study Risk of Bias Tool
- **Assessment**:

- **Recruitment**: Feasibility was well-documented. Eight families were recruited, and there was no significant loss to follow-up, which indicates strong recruitment success for a pilot study.
- **Intervention Delivery**: The My Baby's Sleep (MBS) intervention was delivered as planned in most cases, although some sessions were adjusted due to scheduling conflicts. However, over 46% of the sessions occurred outside the recommended time frames due to logistical barriers.
- **Data Collection**: Surveys were efficiently collected with minimal missing data. There was a reported issue of repetitive survey questions, which may have caused participant fatigue.
- **Outcome Measures**: Self-reported data on engagement and satisfaction, with qualitative interviews and quantitative ratings. There were no objective measures of sleep behavior changes, increasing reliance on subjective reports.
- **Missing Data**: Minimal missing data; participants completed most intervention components and provided sufficient data for feasibility assessment.
- **Overall**: Low risk of bias in terms of feasibility and acceptability. However, the reliance on self-reported data and some deviations from intended intervention timing are noted as minor concerns.

9. Dowling et al. (2018) - ***Cohort Study***

- **Tool**: Newcastle-Ottawa Scale (NOS)
- **Assessment**:
- **Selection Bias**: Mothers of preterm infants were recruited from a NICU, with clear inclusion criteria such as gestational age <37 weeks. This study targeted a well-defined and high-risk population.
- **Comparability**: The study did not have a comparison group, limiting its ability to adjust for potential confounders. However, the study employed repeated measures, allowing comparison within individuals over time.
- **Outcome Assessment**: The primary outcome was adherence to safe sleep practices post-discharge, assessed through surveys. While

informative, this introduces a potential reporting bias as the mothers may not fully adhere to practices reported.

- **Follow-up Adequacy**: Follow-up at four weeks post-discharge was completed by 53% of mothers, which indicates a moderate level of attrition but is reasonable for a cohort study in this context.
- **Overall**: Moderate risk of bias, primarily due to the lack of a comparison group and reliance on self-reported adherence to safe sleep practices.

10. Mathews et al. (2018) - *Randomized Controlled Trial (RCT)*

- **Tool**: Cochrane Risk of Bias Tool (RoB 2)
- **Assessment**:
- **Randomization Process**: The randomization process was clearly described and appropriate for a trial involving 1194 mothers.
- **Deviations from Intended Interventions**: No significant deviations. The intervention group received enhanced messaging about suffocation prevention, and the control group received standard safe sleep advice. Both groups followed the intervention as intended.
- **Missing Outcome Data**: A significant number of participants (53.4%) completed all interviews. This attrition rate is a concern, but follow-up analyses showed that those who completed the study were not systematically different from those who dropped out.
- **Measurement of Outcome**: Outcomes were measured through self-reported surveys, introducing potential response bias, particularly related to socially desirable responses.
- **Selective Reporting**: No evidence of selective reporting. All pre-specified outcomes were reported, and results were consistent across follow-up points.
- **Overall**: Moderate risk of bias due to high attrition and reliance on self-reported data, but the study design was robust, and key outcomes were adequately reported.

11. Brashears et al. (2020) - *Quality Improvement Project*

- **Tool**: Standards for QUality Improvement Reporting Excellence (SQUIRE) Guidelines

- **Assessment**:
    - **Problem Definition**: The authors identified an important gap in infant sleep safety screening and education in pediatric primary care settings. The project aimed to improve screening forms and provide targeted education on safe sleep practices.
    - **Context**: The study was conducted in Oklahoma, where the infant mortality rate was notably higher than national averages, providing strong justification for this quality improvement intervention.
    - **Intervention**: The updated screening form, based on PRAMS questions, was implemented at pediatric well-child checks (WCCs) for infants aged 0–6 months. Follow-up callbacks were conducted two weeks after each WCC to reinforce education and assess any behavior changes.
    - **Study Design**: The intervention was well-documented, with clear inclusion criteria and procedures for screening and callbacks. Data were collected through electronic health records (EHR), and caregivers received a booklet titled *Sleep Baby, Safe and Snug* to reinforce safe sleep practices.
    - **Outcome Measures**: The primary outcome was the identification of unsafe sleep practices, assessed at the WCC and follow-up callback. A statistically significant increase in documentation of unsafe practices (55.8% post-intervention vs 3.7% pre-intervention) indicates that the new screening tool was effective in identifying unsafe behaviors.
    - **Missing Data**: There was minimal missing data, with only one screening form not documented, reflecting strong adherence to the intervention protocol.
    - **Sustainability**: The authors acknowledged limitations, including the possibility of caregivers underreporting unsafe practices after receiving education. However, the use of callbacks and follow-ups helped address this challenge.
    - **Discussion**: The results demonstrated the importance of using standardized, evidence-based tools like PRAMS to improve screening and education around safe sleep. The authors made relevant recommendations for further improvements, such as training staff to handle follow-up calls.

- **Overall**: The quality improvement project effectively met its goals, with clear documentation of the intervention and its impact on screening and education practices. The study presents low risk of bias, with robust data collection and analysis processes.

12. **Goodstein et al. (2015) -** ***Quasi-experimental Study***

- **Tool**: Risk of Bias in Non-Randomized Studies of Interventions (ROBINS-I)
- **Assessment**:
  - **Bias due to Confounding**: The study aimed to evaluate a comprehensive hospital-based infant sleep safety (ISS) program. Confounding variables were minimized by using a pre- and post-intervention survey at hospital discharge and 4-month follow-up. However, there was no random allocation, leading to moderate bias from confounding factors.
  - **Bias in Selection of Participants**: Participants were recruited in the hospital after completing the education program, and consent was obtained. Selection was not random, so there is some risk of bias in how the sample was chosen, particularly given the focus on families attending specific hospitals.
  - **Bias in Classification of Interventions**: The intervention (ISS education) was consistently applied across participants. Differences between intervention sites, such as mandatory versus voluntary DVD viewing, were clearly documented, indicating a low risk of misclassification bias.
  - **Bias Due to Missing Data**: At hospital discharge, 1,092 surveys were analyzed, but at follow-up, only 490 surveys were completed. This represents a moderate attrition rate, introducing a risk of bias due to missing data. The authors addressed this limitation by comparing the study population with national benchmarks, which partially mitigates the bias.
  - **Bias in Measurement of Outcomes**: The outcomes were measured through self-reported surveys, which may introduce bias due to social desirability or recall inaccuracies. However, the use of standardized surveys minimized the risk of significant outcome measurement bias.

- **Bias in Selection of Reported Results**: There was no evidence of selective reporting, as all pre-specified outcomes related to infant sleep safety were reported in the results.

- **Overall**: The study presents a moderate risk of bias, primarily due to the non-randomized design and reliance on self-reported data. Attrition also contributed to potential bias, but overall, the study was well-executed.

13. Leichman et al. (2020) - *mHealth Intervention Study*

- **Tool**: Cochrane Risk of Bias Tool (RoB 2)
- **Assessment**:
  - **Randomization Process**: This study assessed the effectiveness of an mHealth sleep intervention for infants, delivered through a smartphone app. Participants were not randomly assigned to groups, as this was a real-world study. This introduces a risk of bias due to non-random allocation.
  - **Deviations from Intended Interventions**: The intervention was delivered via a publicly available mobile app, with no significant deviations from the intended protocol. However, the lack of control over participants' adherence to the app's recommendations is a potential source of bias.
  - **Missing Outcome Data**: The study followed participants over a 4-28 day period, with no significant issues reported in missing data. All participants who used the app twice were included in the analysis.
  - **Measurement of Outcomes**: Sleep parameters were measured through self-reported data from the app, introducing potential bias due to subjective reporting. While the study used validated tools like the Brief Infant Sleep Questionnaire–Revised (BISQ-R), the lack of objective sleep measurements (e.g., actigraphy) is a limitation.
  - **Selective Reporting**: There is no evidence of selective reporting. All pre-specified outcomes, such as sleep onset latency and night waking frequency, were reported in detail.

- **Overall**: Moderate risk of bias due to the non-randomized design and reliance on self-reported outcomes. The real-world nature of the study adds value, but the lack of a control group limits the strength of the conclusions.

14. Martins et al. (2018) - ***Longitudinal Experimental Study***

- **Tool**: Newcastle-Ottawa Scale (NOS)
- **Assessment**:
- **Selection Bias**: The study was well-designed with clear inclusion criteria, focusing on mothers of newborns in a tertiary hospital in Portugal. The selection was random based on weekday assignments for the intervention and control groups.
- **Comparability**: There were no significant differences in baseline characteristics between the intervention and control groups, and adjustments were made for maternal age, education, and race.
- **Outcome Assessment**: The main outcomes (infants' sleep habits) were assessed via questionnaires administered at 1, 2, 4, and 6 months, with strong follow-up rates.
- **Follow-up Adequacy**: The study achieved an adequate follow-up rate, with most mothers completing at least three questionnaires during the 6-month follow-up period.
- **Overall**: Low risk of bias, with a well-structured intervention and robust follow-up. The study design minimizes the risk of selection and attrition biases.

15. Moon et al. (2017) - ***Randomized Controlled Trial (RCT)***

- **Tool**: Cochrane Risk of Bias Tool (RoB 2)
- **Assessment**:
- **Randomization Process**: The randomization process was well-described and performed at 16 hospitals, with allocation concealment ensured.
- **Deviations from Intended Interventions**: The mHealth intervention was consistently delivered as planned, with minimal deviation.

- **Missing Outcome Data**: The follow-up rate was 78.9%, which is acceptable for this type of trial. However, some attrition occurred, but it was not significantly biased between groups.
- **Measurement of Outcomes**: The outcomes were measured through self-reported adherence to safe sleep practices, introducing potential bias. However, adjustments were made for confounders.
- **Selective Reporting**: No evidence of selective reporting.
- **Overall**: Low to moderate risk of bias, with a robust intervention but some reliance on self-reported data.

16. Nabaweesi et al. (2020) - ***Quasi-experimental Study***

- **Tool**: Risk of Bias in Non-Randomized Studies of Interventions (ROBINS-I)
- **Assessment**:
- **Bias Due to Confounding**: The study assessed sleep environments using smartphone technology, targeting teen mothers. The lack of randomization introduces potential confounding bias.
- **Bias in Selection of Participants**: Participants were selected via convenience sampling, increasing the risk of selection bias.
- **Bias in Classification of Interventions**: Intervention (training in using smartphones to capture sleep environments) was consistently applied.
- **Bias Due to Missing Data**: A significant proportion of mothers completed the home visit and provided photographs, with low missing data.
- **Bias in Measurement of Outcomes**: The use of both photographs and direct observations minimizes bias, although reliance on self-reports could introduce some bias.
- **Selective Reporting**: No selective reporting detected.
- **Overall**: Moderate risk of bias, mainly due to non-randomized design and convenience sampling.

17. Paul et al. (2016) - ***Randomized Controlled Trial (RCT)***

- **Tool**: Cochrane Risk of Bias Tool (RoB 2)
- **Assessment**:
- **Randomization Process**: The study employed a clear randomization process with stratification based on birth weight and intended feeding mode.
- **Deviations from Intended Interventions**: No significant deviations from the intended intervention (responsive parenting vs control).
- **Missing Outcome Data**: The follow-up rate was high, with 90.7% of dyads completing the 1-year visit, indicating low risk of attrition bias.
- **Measurement of Outcomes**: The study used validated tools, including the Brief Infant Sleep Questionnaire, minimizing bias in outcome measurement.
- **Selective Reporting**: No evidence of selective reporting.
- **Overall**: Low risk of bias, with a well-executed intervention and robust follow-up.

18. Salm Ward et al. (2018) - ***Cohort Study***

- **Tool**: Newcastle-Ottawa Scale (NOS)
- **Assessment**:
- **Selection Bias**: The study targeted low-income women, providing clear inclusion criteria related to pregnancy stage and financial need.
- **Comparability**: The study did not have a control group but compared pre- and post-intervention outcomes within individuals, adjusting for confounders.
- **Outcome Assessment**: Knowledge and practices were assessed using pre- and post-test surveys, with a follow-up survey to measure changes in behavior.
- **Follow-up Adequacy**: Adequate follow-up, with 132 participants completing the pre- and post-tests, and 76 participants completing the follow-up survey.
- **Overall**: Low to moderate risk of bias, with strong follow-up and clear outcomes but lacking a comparison group.

19. Santos et al. (2019) - ***Randomized Controlled Trial (RCT)***

- **Tool**: Cochrane Risk of Bias Tool (RoB 2)
- **Assessment**:
  - **Randomization Process**: Adequately randomized with clear methods, using opaque envelopes for allocation concealment.
  - **Deviations from Intended Interventions**: There were no significant deviations. The intervention was conducted as intended, involving counseling on sleep habits at home visits.
  - **Missing Outcome Data**: Some attrition occurred, but it was balanced between the intervention and control groups, minimizing bias due to missing data.
  - **Measurement of Outcomes**: The study employed actigraphy and sleep diaries, providing objective and subjective measures of sleep duration. This dual approach reduces measurement bias.
  - **Selective Reporting**: All pre-specified outcomes were reported, including actigraphy data and sleep duration.
- **Overall**: Low risk of bias, with robust methodology and minimal deviations from protocol.

20. Sweeney et al. (2020) - ***Controlled Pilot Study***

- **Tool**: Cochrane Risk of Bias Tool (RoB 2)
- **Assessment**:
  - **Randomization Process**: The study did not use random allocation but assigned participants based on recruitment site, introducing potential bias due to non-random allocation.
  - **Deviations from Intended Interventions**: The intervention was delivered as planned, with prenatal and postnatal support sessions. Follow-up calls were also made, although not all were completed, leading to some minor deviations.

- **Missing Outcome Data**: Low attrition rates, with all participants completing at least two follow-up calls, suggesting low risk of bias due to missing data.
- **Measurement of Outcomes**: Both maternal and infant sleep outcomes were measured via actigraphy and sleep diaries, providing reliable objective and subjective data.
- **Selective Reporting**: No evidence of selective reporting. All planned outcomes were reported, including maternal sleep quantity and infant sleep consolidation.

- **Overall**: Moderate risk of bias due to non-randomized design, but outcomes were measured reliably.

21. Thompson et al. (2018) - ***Randomized Controlled Trial (RCT)***

- **Tool**: Cochrane Risk of Bias Tool (RoB 2)
- **Assessment**:
  - **Randomization Process**: The study employed a clear randomization process using a computer-generated function. Participants were randomly assigned to two groups in blocks of 25.
  - **Deviations from Intended Interventions**: No major deviations were reported. The home visiting program was delivered as intended, focusing on infant activity and sleep behaviors.
  - **Missing Outcome Data**: The study had a high retention rate (85%), suggesting low risk of bias due to attrition.
  - **Measurement of Outcomes**: Sleep behaviors were assessed using maternal reports, which could introduce bias, but validated tools were used, and compliance with sleep recommendations was tracked over time.
  - **Selective Reporting**: There was no evidence of selective reporting.
- **Overall**: Low risk of bias, with strong adherence to protocol and reliable outcome measurement

22. Abuhammad et al. (2024) - ***Quasi-experimental Study***

- **Tool**: Risk of Bias in Non-Randomized Studies of Interventions (ROBINS-I)
- **Assessment**:
  - **Bias Due to Confounding**: The study aimed to assess the impact of an educational intervention on mothers' knowledge of infant sleep. The non-random allocation of participants introduces a moderate risk of confounding.
  - **Bias in Selection of Participants**: Convenience sampling was used, and mothers chose their group, leading to a higher risk of selection bias.
  - **Bias in Classification of Interventions**: The classification of the educational intervention was consistent across groups, but variability in the mode of delivery (online vs. in-person) could have influenced results.
  - **Bias Due to Missing Data**: The study had minimal missing data, with both groups completing the pre- and post-tests. Attrition was low, indicating a low risk of bias due to missing data.
  - **Bias in Measurement of Outcomes**: Self-reported knowledge and attitudes were used, which introduces potential bias. However, the use of validated questionnaires helps mitigate this risk.
  - **Selective Reporting**: No evidence of selective reporting. All planned outcomes were reported in the results.
- **Overall**: Moderate risk of bias due to the non-randomized design and reliance on self-reported data, but the intervention was consistently delivered and data collection was comprehensive

23. Huber et al. (2024) - ***Mixed Methods Study***

- **Tool**: Mixed Methods Appraisal Tool (MMAT)
- **Assessment**:
  - **Qualitative Component**: Key informant interviews were used to collect data on community-level infant safe sleep (ISS) and breastfeeding promotion. The qualitative framework was well-

described, employing hermeneutical phenomenology. Themes were consistently coded, and triangulation was employed to validate findings.

  - **Quantitative Component**: State-level data from PRAMS and OPAS were used to assess infant safe sleep and breastfeeding practices. The data were descriptively analyzed, and geographic and racial/ethnic disparities were explored in detail.
  - **Integration of Qualitative and Quantitative Data**: The study employed an embedded integration approach, linking qualitative insights with quantitative state-level indicators. The integration of data was appropriate, and the results were presented in a clear, cohesive manner.
  - **Sampling**: The sampling strategy for the qualitative component was appropriate for the research questions, but the limited number of key informants (n=7) may reduce the generalizability of the findings. The quantitative component used robust population-level data, enhancing the study's external validity.
  - **Bias in Reporting**: The study transparently reported both qualitative and quantitative findings, with no evidence of selective reporting.
- **Overall**: Low risk of bias, with robust integration of qualitative and quantitative data. The mixed methods design was well-executed
